# Human Object-Similarity Judgments Reflect and Transcend the Primate-IT Object Representation

**DOI:** 10.3389/fpsyg.2013.00128

**Published:** 2013-03-22

**Authors:** Marieke Mur, Mirjam Meys, Jerzy Bodurka, Rainer Goebel, Peter A. Bandettini, Nikolaus Kriegeskorte

**Affiliations:** ^1^Section on Functional Imaging Methods, Laboratory of Brain and Cognition, National Institute of Mental Health, National Institutes of HealthBethesda, MD, USA; ^2^Department of Cognitive Neuroscience, Faculty of Psychology and Neuroscience, Maastricht UniversityMaastricht, Netherlands; ^3^Medical Research Council, Cognition and Brain Sciences UnitCambridge, UK; ^4^Functional Magnetic Resonance Imaging Facility, National Institute of Mental Health, National Institutes of HealthBethesda, MD, USA

**Keywords:** object perception, vision, neuronal representation, fMRI, representational similarity analysis, human, primate

## Abstract

Primate inferior temporal (IT) cortex is thought to contain a high-level representation of objects at the interface between vision and semantics. This suggests that the perceived similarity of real-world objects might be predicted from the IT representation. Here we show that objects that elicit similar activity patterns in human IT (hIT) tend to be judged as similar by humans. The IT representation explained the human judgments better than early visual cortex, other ventral-stream regions, and a range of computational models. Human similarity judgments exhibited category clusters that reflected several categorical divisions that are prevalent in the IT representation of both human and monkey, including the animate/inanimate and the face/body division. Human judgments also reflected the within-category representation of IT. However, the judgments transcended the IT representation in that they introduced additional categorical divisions. In particular, human judgments emphasized human-related additional divisions between human and non-human animals and between man-made and natural objects. hIT was more similar to monkey IT than to human judgments. One interpretation is that IT has evolved visual-feature detectors that distinguish between animates and inanimates and between faces and bodies because these divisions are fundamental to survival and reproduction for all primate species, and that other brain systems serve to more flexibly introduce species-dependent and evolutionarily more recent divisions.

## Introduction

How does our percept of the similarity of two objects arise from our internal representation of the objects? One influential theory holds that perceived similarity can be explained on the basis of the distance between the objects in a conceptual space (e.g., Gärdenfors, 2004). A conceptual space can be seen as analogous to the spatial environment that we live in: in both the location of an object is determined by its positions on a set of dimensions. The difference lies in the dimensions that define the space: for our spatial environment, the location of an object can be specified by three spatial coordinates (*x*, *y*, and *z* dimensions); for a conceptual space, the dimensions can be any object properties, including perceived color, shape, or semantic category. The location of a perceived object in a conceptual space is interpreted as the mental representation of that object. Distances between object representations inform us about their relationships: the greater the distance, the greater the perceived dissimilarity. Perceived similarity can be estimated by asking observers to make explicit object-similarity judgments.

How the perceived similarity of two objects can be explained on the basis of their mental representation has long been of interest to philosophers, mathematicians, and psychologists (e.g., Carnap, 1928/1967; Shepard, 1958; Torgerson, 1958; Rosch et al., 1976; Tversky, 1977; Edelman, 1998). The geometrical model of the mental representation described above (e.g., Shepard, 1958; Torgerson, 1958; Edelman, 1998) can account for a great variety of empirical findings and the most recent versions (e.g., Gärdenfors, 2004) also account for phenomena, such as context dependence, that were initially thought to be difficult to accommodate (Goodman, 1972; Tversky, 1977; for a recent review, see Decock and Douven, 2011). Importantly, the geometrical model enables a direct comparison between brain representational similarity and similarity judgments. In keeping with the concept of distance in a representational space, we describe judgments and brain representations in terms of dissimilarities, rather than similarities. We study correlations between representational dissimilarity matrices (RDMs) within the framework of representational similarity analysis (RSA, Kriegeskorte et al., 2008a) to quantitatively compare brain, behavior, and computational models.

Object representations are thought to be implemented in the brain by means of population codes (e.g., Sanger, 2003). If the neurons represent the dimensions of some conceptual space, then the distances in neuronal pattern space are identical to the conceptual distances. Neuronal recordings and functional magnetic resonance imaging (fMRI) both provide only very impoverished samples of a neuronal population code. With recordings we are missing most of the cells. With fMRI, each voxel reflects a local spatiotemporal average of neuronal activity. In either case, we are sampling a subset of the dimensions of the neuronal response space. However, the representational distance in our sample can be interpreted as an estimate of the representational distance in the neuronal population code (e.g., Kiani et al., 2007; Kriegeskorte et al., 2008b). This line of thought has become increasingly popular in recent years (Edelman et al., 1998; Haxby et al., 2001; McClelland and Rogers, 2003; Kriegeskorte and Kreiman, 2011).

Multiple studies have shown that distributed activity patterns in human inferior temporal (IT) cortex – a large region of object-selective cortex located in the ventral visual stream – contain information about category membership of visual objects (Haxby et al., 2001; Cox and Savoy, 2003). These results are broadly consistent with earlier findings by Edelman et al. (1998), who pioneered the application of geometrical models of shape similarity to brain data and showed initial evidence for clustering by category. Grouping individual real-world objects on the basis of the similarity of the activity patterns they elicit in IT reveals clusters corresponding to well-known object categories, including animate and inanimate objects and, within the animates, faces, and bodies (Kiani et al., 2007; Kriegeskorte et al., 2008b). Major category clusters (e.g., animates) contain smaller clusters (e.g., faces and bodies), suggesting a hierarchical organization. The categorical divisions are strikingly similar between human and monkey IT (mIT) and, importantly, not accounted for by a range of computational models of low- and intermediate complexity features (Kriegeskorte et al., 2008b).

The presence of hierarchically organized clusters that correspond to well-known object categories parallels earlier findings on human categorization behavior by Rosch et al. (1976), who introduced the concept of superordinate (e.g., animate objects), basic (e.g., faces), and subordinate categories (e.g., female faces). This parallel suggests that IT, which is thought to be at the interface of perception and cognition, might be the neuronal substrate for the mental representations giving rise to object-similarity judgments. In line with this idea, several studies have suggested a relationship between perceived similarity and activity-pattern similarity in primate object-selective cortex for abstract and computer-generated visual shapes (Edelman et al., 1998; Op de Beeck et al., 2001, 2008; Kayaert et al., 2005; Haushofer et al., 2008). However, these studies have not thoroughly investigated the mental similarity representation of real-world object images and its relation to the inherently categorical IT representation. Do human object-similarity judgments reflect the IT object space, including its hierarchy of category clusters?

**Figure 1:**
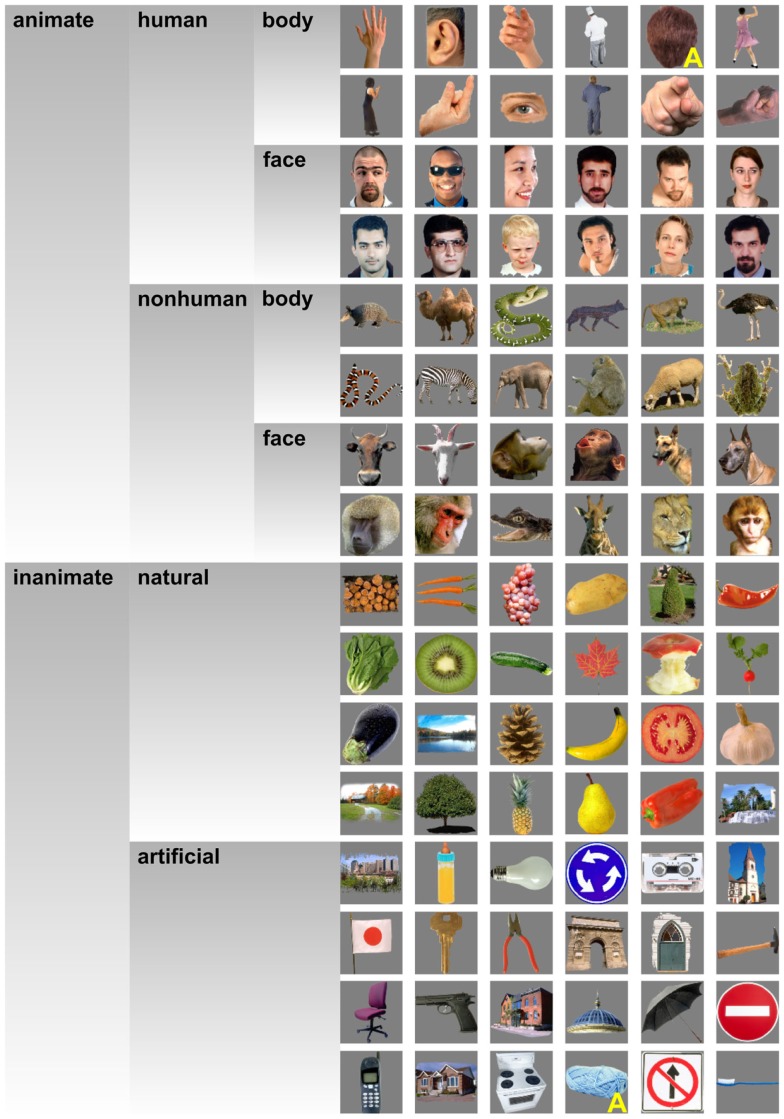
**Stimuli**. This figure shows the object images that we presented to our subjects. Two stimuli were described as ambiguous by several of our subjects during debriefing. These stimuli (back of a human head, knitting wool) are marked with a yellow “A.” This figure is adopted from Kriegeskorte et al. (2008b).

**Figure 2:**
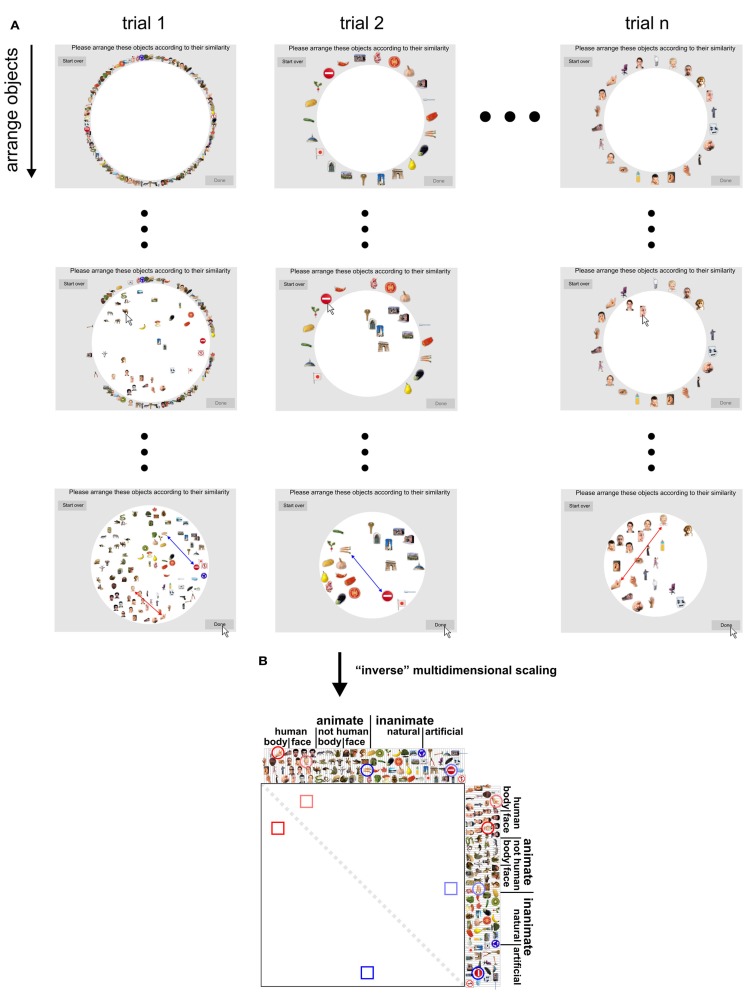
**Dissimilarity judgments by multi-arrangement (MA)**. **(A)** Dissimilarity judgments were acquired using a novel MA method, which allows efficient and subject-tailored acquisition of perceived similarity for large sets of objects. Subjects were asked to arrange the objects according to their similarity, using mouse drag-and-drop on a computer display. Perceived similarity was communicated by adjusting the distances between the objects: objects perceived as similar were placed close together; objects perceived as dissimilar were placed further apart. The upper panel of the figure shows screenshots taken at different moments during the acquisition of the dissimilarity judgments for one subject. Columns correspond to trials and rows show object arrangements over time, running from the start (first row) to the end of each trial (final arrangement, last row). The first trial contained all object images; subsequent trials contained subsets of images that were adaptively selected to optimally estimate perceived similarity for each subject. The black dots represent not-shown arrangements during a trial (small dots) and not-shown trials (large dots). **(B)** Once acquisition of the dissimilarity judgments was completed, inter-object distances of the final trial arrangements were combined over trials by rescaling and averaging to yield a single dissimilarity estimate for each object pair. Conceptually, this step can be seen as “inverse” multidimensional scaling, since it combines several lower-dimensional (2D) similarity representations into one higher-dimensional similarity representation. This process is shown for two example objects pairs: a boy’s face and a hand (red), and carrots and a stop sign (blue). Their single-trial dissimilarity estimates (arrows) are combined into a single dissimilarity estimate, which is placed at the corresponding entry of the RDM (lower panel). Mirror-symmetric entries are indicated by lighter colors.

**Figure 3:**
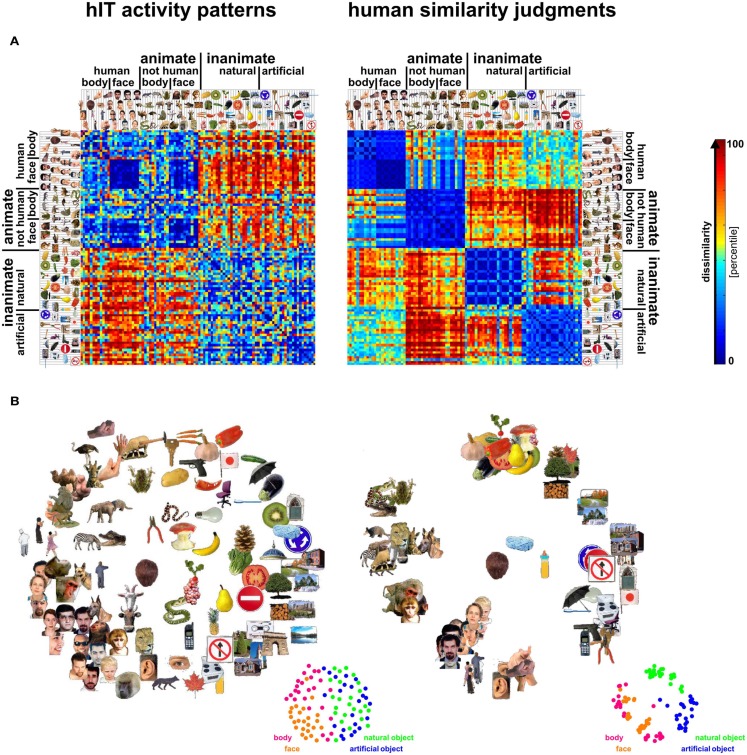
**Representational dissimilarity matrices (RDMs) and MDS arrangements for human IT and judgments**. Human IT activity patterns and human similarity judgments both show an inherently categorical representation of real-world object images with an animate/inanimate top-level division. At the same time, the similarity judgments show additional categorical divisions and stronger clustering than the hIT similarity representation. **(A)** RDMs based on hIT activity patterns and human similarity judgments. Each RDM is based on data from multiple subjects (4 and 16, respectively), averaged at the level of the dissimilarities. Each entry of a matrix represents hIT activity-pattern dissimilarity (1-*r*, where *r* is Pearson correlation coefficient; 316 most visually responsive bilateral hIT voxels defined using independent data) or judged dissimilarity (relative Euclidean distance as measured by the MA method) for a pair of objects. The matrices were independently transformed into percentiles (see color bar). **(B)** Multidimensional scaling (MDS; criterion: metric stress) was used to visualize the hIT and judgment similarity representations of the 96 real-world object images. Distances between images reflect the dissimilarities that are shown in the RDMs in **(A)**: images that elicited similar activity patterns or that were judged as similar are placed close together; images that elicited dissimilar activity patterns or were judged as dissimilar are placed further apart.

**Figure 4:**
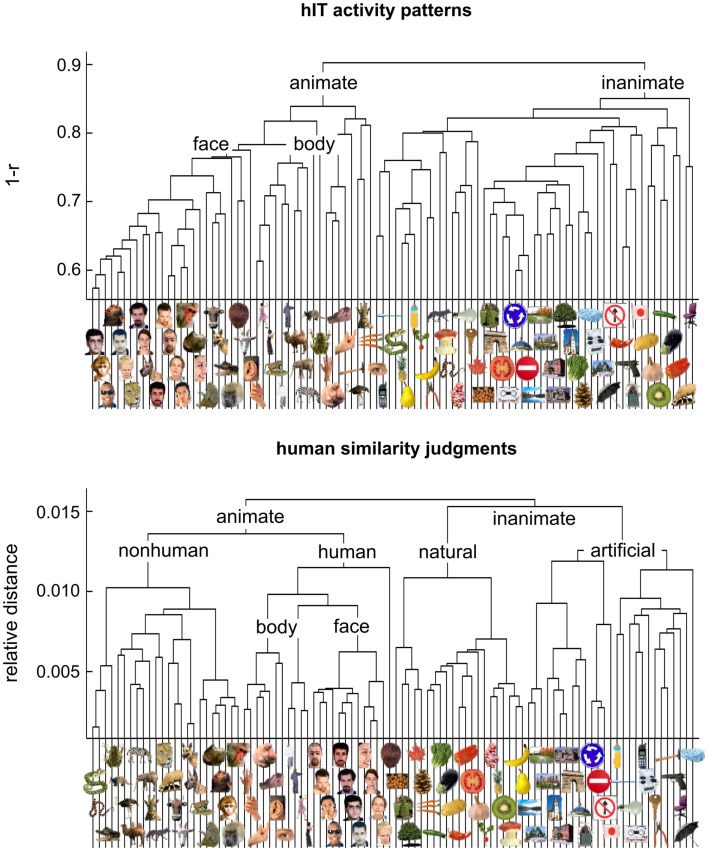
**Hierarchical clustering for human IT and human judgments**. hIT object-activity patterns have been shown to cluster according to natural categories (top panel) (Kriegeskorte et al., 2008b). In order to assess whether human object-similarity judgments show a similar categorical structure, we performed hierarchical cluster analysis on the similarity judgments (bottom panel). Hierarchical cluster analysis starts with single-image “clusters” and successively combines the two clusters closest to each other to form a hierarchy of clusters. The vertical height of each horizontal link reflects the average dissimilarity between the stimuli of two linked subclusters. hIT activity-pattern dissimilarity was measured as 1-*r* (where *r* is Pearson correlation coefficient), judged dissimilarity was measured as relative Euclidean distance (using the MA method). Text labels indicate the major clusters. Both hIT activity patterns and human similarity judgments cluster the objects according to natural categories and show a top-level animate/inanimate division. However, the human similarity judgments introduce additional categorical divisions.

**Figure 5:**
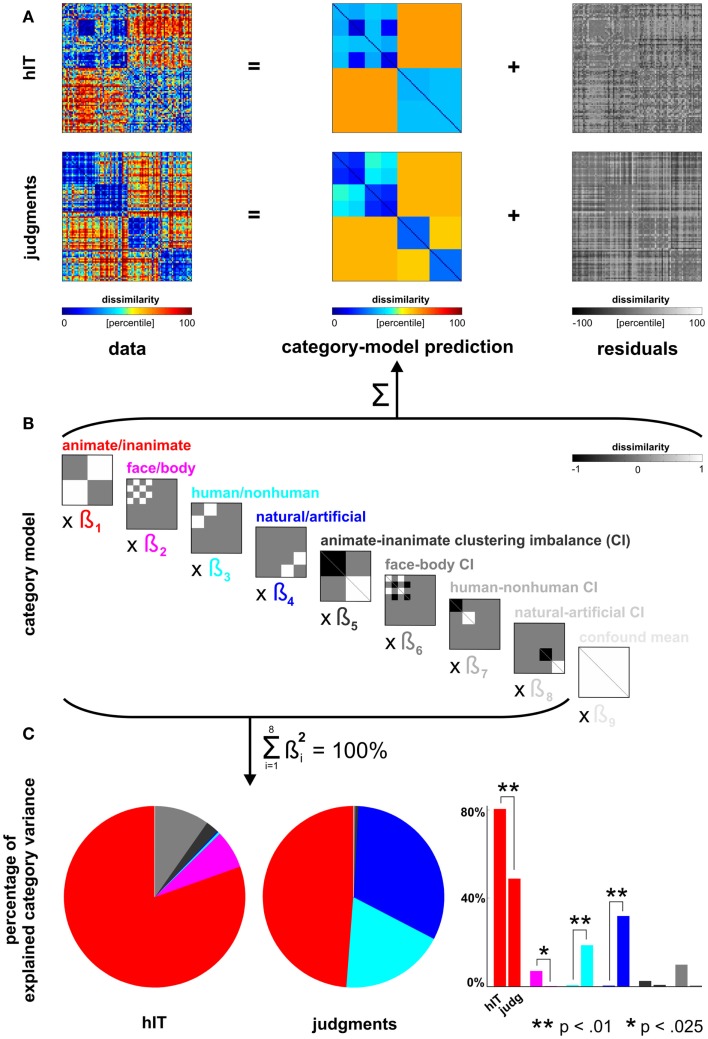
**Human dissimilarity judgments emphasize additional categorical divisions not present in human IT**. **(A)** We decomposed the dissimilarity matrices for hIT and judgments into two additive components, reflecting the category-related dissimilarity variance and non-category-related dissimilarity variance (i.e., within-category dissimilarities and noise). **(B)** The decomposition was performed by fitting a linear model with multiple predictor dissimilarity matrices, each reflecting a categorical division (red, magenta, cyan, blue) or an imbalance between average within-category dissimilarities of two categories (e.g., average within-animate dissimilarity < average within-inanimate dissimilarity). We fitted the model to the RDMs for hIT and judgments using ordinary-least-squares and estimated the ratio of category-related dissimilarity variance (captured by the model) and non-category-related dissimilarity variance (residuals). We then equated the proportion of residual variance by adding noise to the RDM with smaller proportion residual variance. The judgments had a smaller proportion of residual variance. The judgments matrix shown in A contains the added noise. Equating the residual variance is necessary for valid statistical inference (for details on the noise model and inference, see Materials and Methods). **(C)** We then fitted the model to the residual-equated RDMs and compared hIT and judgments in terms of the percentage of category variance explained by each category division. The animate/inanimate and face/body divisions explained significantly more variance in hIT than in the judgments. The human/non-human and natural/artificial divisions explained significantly more variance in the judgments than in hIT.

**Figure 6:**
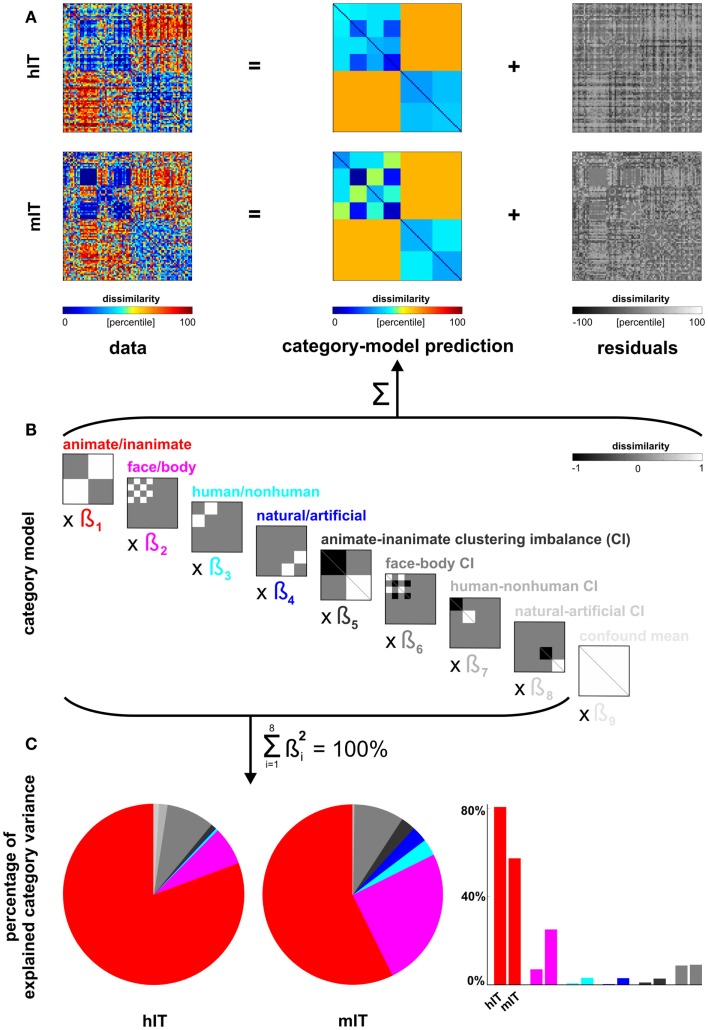
**Categorical divisions in human IT and monkey IT**. We used the linear model from Figure 5 (repeated in **(B)** for convenience) also to compare the IT representations between human and monkey [same data as in Kriegeskorte et al. (2008b) for both species; a more in-depth analysis of the monkey data is Kiani et al. (2007)]. **(A**,**B)** The proportion of residual variance was greater in mIT than hIT. Residual variance was therefore equated by adding noise to the hIT matrix (which is therefore not identical to Figure 5). **(C)** Descriptively, the animate/inanimate and face/body divisions are prominent in both hIT and mIT and the human/non-human and natural/artificial divisions less so. Monkey IT might emphasize the animate/inanimate division less and the face-body division more relative to human IT. However, we could not perform the randomization test of Figure 5 here, because there were only two monkey subjects. For further inferential analyses comparing hIT, mIT, and human judgments, see Figure 7.

**Figure 7:**
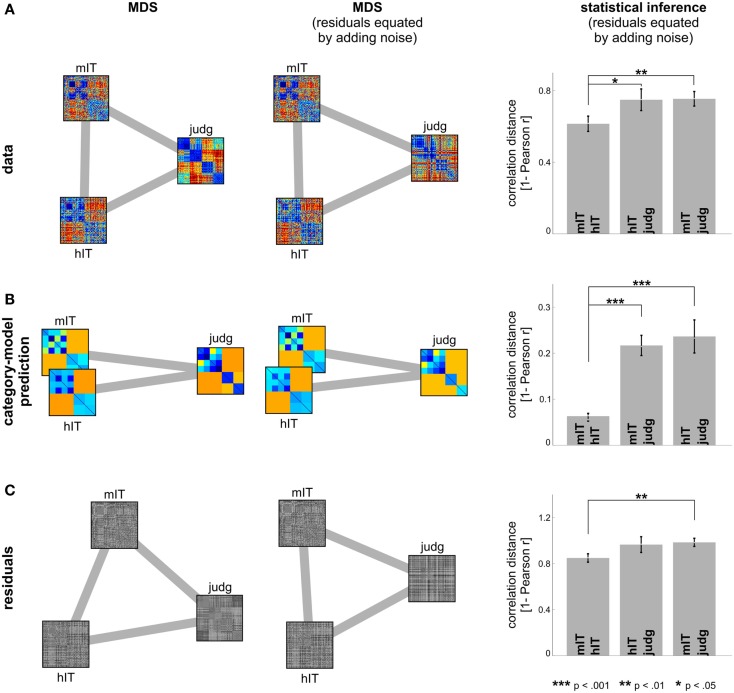
**Human IT and monkey IT are more similar to each other than to human judgments**. **(A)** hIT, mIT, and human judgment RDMs compared in a second-order MDS arrangement (criterion: metric stress; distance measure: 1 – Pearson *r*) before (left) and after (middle) equating the proportion of non-category-related variance by adding dissimilarity noise to the hIT and judgment RDMs. Statistical inference (right, via bootstrapping the stimulus set) indicates that hIT and mIT RDMs are more similar to each other than either of them is to human judgments. **(B)** The same analysis applied to the predicted RDMs of the category-model (Figure 5) suggests that hIT and mIT are very similar in terms of the categorical divisions they emphasize and significantly more similar to each other in this respect than either of them is to human judgments. **(C)** The same analysis applied to the residual RDMs of the category-model shows a weak reflection of the category-model results: hIT and mIT appear slightly more similar to each other than either of them is to the human judgments.

**Figure 8:**
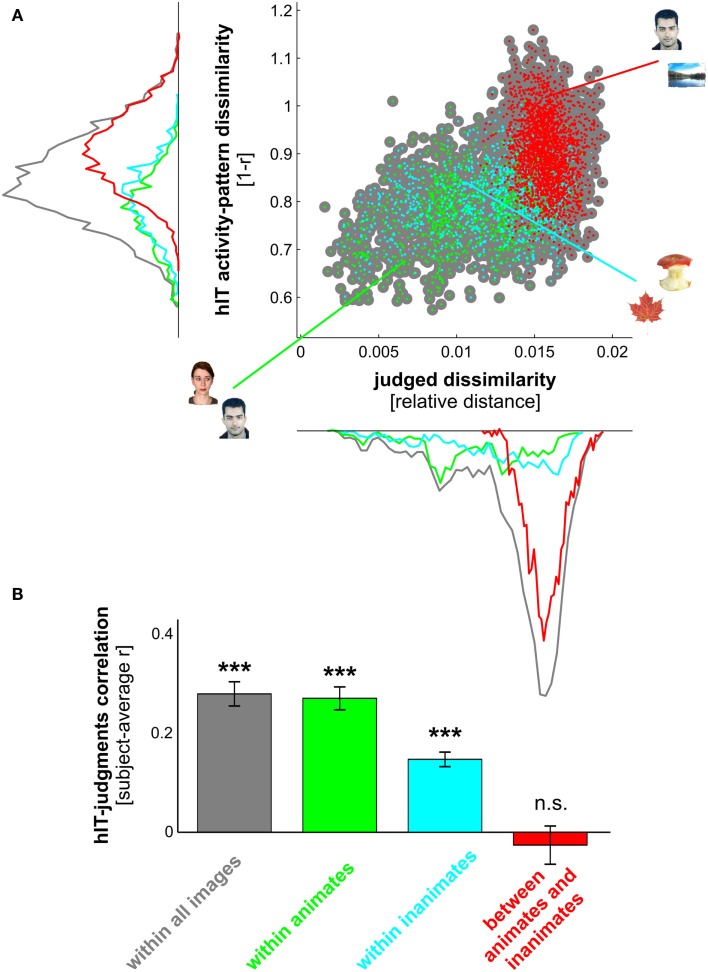
**hIT activity-pattern dissimilarities and judged dissimilarities are significantly correlated within all images and within category subsets of images**. **(A)** Scatter plot of hIT activity-pattern dissimilarities and judged dissimilarities taken from the subject-average RDMs shown in Figure 3A. A dot is placed for each stimulus pair based on its hIT activity-pattern dissimilarity and judged dissimilarity (three example stimulus pairs are shown). The large gray dots represent all possible stimulus pairs (*r* = 0.39, *p* < 0.0001; *r* is Spearman correlation coefficient). The smaller colored dots placed on top of the gray dots code for subsets of images: green dots represent animate object pairs (*r* = 0.34, *p* < 0.0001), cyan dots represent inanimate object pairs (*r* = 0.19, *p* < 0.0001), and red dots represent object pairs consisting of an animate and an inanimate object (*r* = −0.16, *p* < 0.9975). Consistent with the results in Figure 3, the marginal histograms show that both hIT and judged dissimilarities are larger for object pairs that cross the animate-inanimate category boundary (red) than for object pairs that do not cross this boundary (green and cyan). **(B)** To test whether the continuous match between hIT and judged dissimilarities would generalize to the population of similarity judgment subjects, we computed the correlation of each single-subject judgment RDM with the subject-average hIT RDM and tested whether the average of those correlations was significantly larger than zero, using a one-sample *t* test. Bars show the average correlation between hIT and judged dissimilarities across subjects. Error bars show SEM. Asterisks indicate significance (*p* < 0.001).

**Figure 9:**
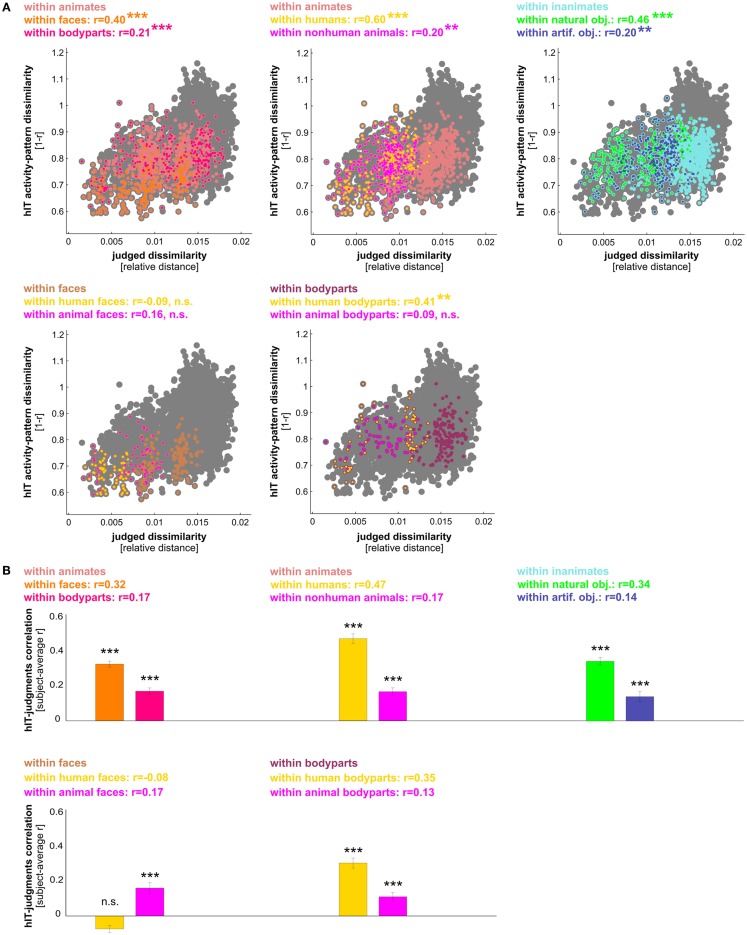
**hIT activity-pattern dissimilarities and judged dissimilarities are significantly correlated within most finer-grained category subsets of images**. **(A)** Scatter plots of hIT and judged dissimilarities taken from the subject-average RDMs in Figure 3A. A dot is placed for each stimulus pair based on its hIT activity-pattern dissimilarity and judged dissimilarity. The large gray dots represent all possible stimulus pairs, the smaller colored dots placed on top of the gray dots code for subsets of images as indicated in the plot legends. Plot legends show Spearman correlation coefficients and associated *p*-values computed with a one-sided stimulus-label randomization test (10,000 randomizations). Asterisks indicate significance (*** = *p* < 0.001, ** = *p* < 0.01). The hIT and judgment similarity structures are significantly correlated within the following subsets of images: faces, bodies, human bodies, humans, non-human animates, natural objects, and artificial objects. This suggests a shared within-category similarity structure. **(B)** The within-category match between hIT activity-pattern dissimilarities and judged dissimilarities generalizes to the population of similarity judgment subjects. We computed the correlation of each single-subject similarity judgment RDM with the subject-average hIT RDM and tested whether the average of those correlations was significantly larger than zero, using a one-sample *t* test. Bars show the average correlation between hIT and judged dissimilarities across subjects. Error bars show SEM. Asterisks indicate significance (*p* < 0.001).

**Figure 10:**
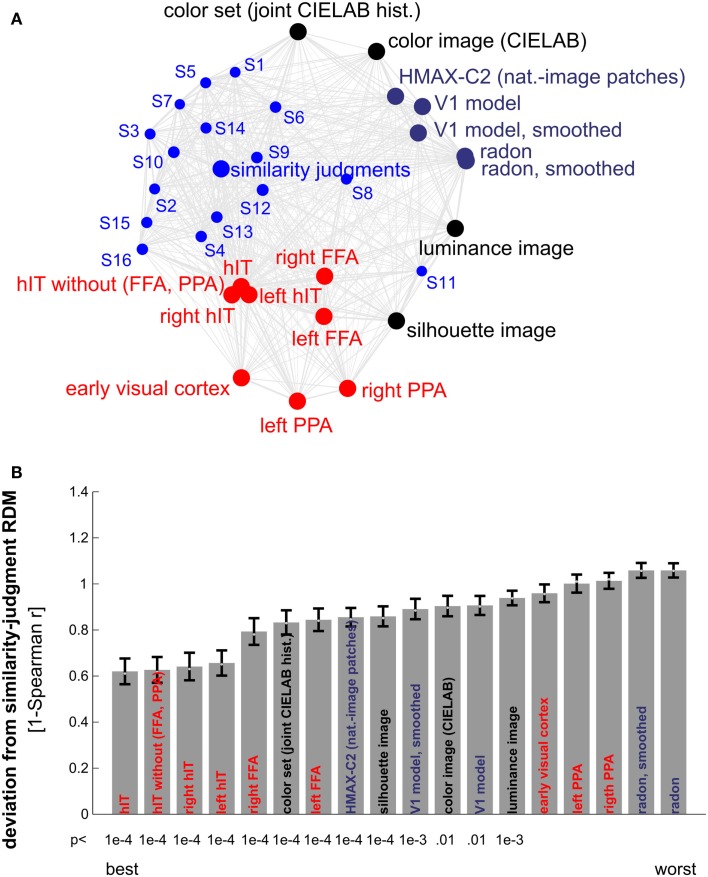
**Similarity judgments’ match to brain and model representations**. **(A)** Multidimensional scaling of similarity representations (criterion: metric stress, distance measure: 1-*r*, where *r* is Spearman correlation coefficient). The MDS plot visualizes the relationships between multiple RDMs simultaneously. Text-label colors indicate the type of similarity representation: red indicates brain-activity, blue indicates human similarity judgments, black indicates simple computational models, and gray/blue indicates complex computational models. Single-subject similarity judgment RDMs are shown as well (smaller font). The gray connections between the RDMs reflect the inevitable distortions induced by arranging the higher-dimensional similarity representations in a lower-dimensional space (2D). **(B)** Match bars for several brain regions and models showing their deviation from the subject-average similarity judgment RDM. The deviation is measured as 1 − Spearman correlation between RDMs. Text color encodes the type of representation as in **(A)**. Error bars indicate the standard error of the deviation estimate. The standard error was estimated as the standard deviation of 100 deviation estimates obtained from bootstrap resamplings of the condition set. The *p*-value below each bar indicates whether the associated RDM is significantly related to the similarity judgment RDM (stimulus-label randomization test, 10,000 randomizations). hIT is the best match to the similarity judgments.

**Figure 11:**
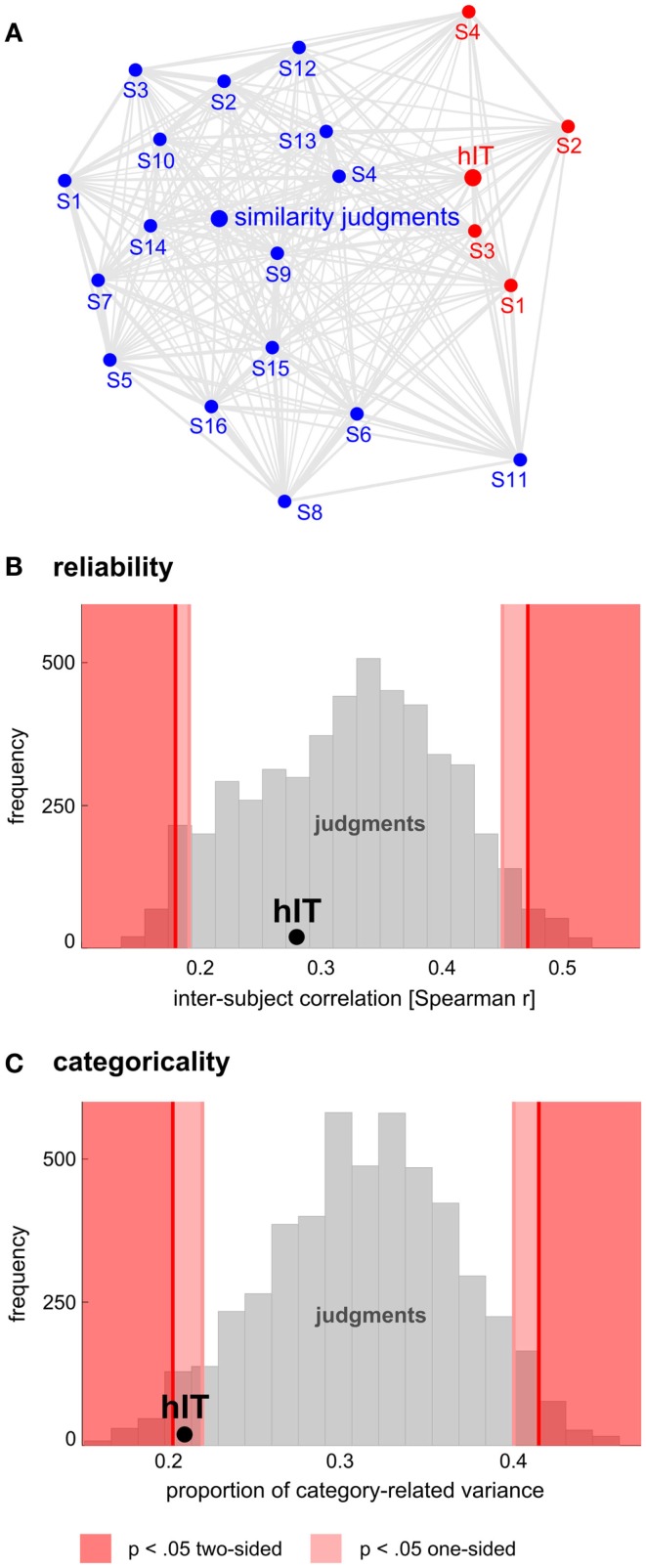
**Human judgments show similar reliability but stronger categoricality than human IT**. **(A)** Multidimensional scaling of single-subject similarity representations (criterion: metric stress, distance measure: 1-*r*, where *r* is Spearman correlation coefficient). The MDS plot visualizes the relationships between multiple RDMs simultaneously. Text-label colors indicate the type of similarity representation: red indicates human IT, blue indicates human similarity judgments. Subject-average RDMs are shown in larger font. The gray connections between the RDMs reflect the inevitable distortions induced by arranging the higher-dimensional similarity representations in a lower-dimensional space (2D). Visual inspection of the MDS plot suggests that variability across subjects is similar for judgments and hIT. **(B)** This panel shows inter-subject reliability for hIT and judgments. We estimated inter-subject reliability as the average pairwise inter-subject RDM correlation (Spearman *r*), using sets of four subjects (one set for hIT; 5,000 randomly selected subsets for the judgments). The hIT reliability falls well within the judgment distribution, indicating that hIT and judgments do not significantly differ in terms of reliability. **(C)** This panel shows categoricality for hIT and judgments. We estimated categoricality as the proportion of dissimilarity variance explained by the category-model (Figure 5B), averaged across sets of four subjects (one set for hIT; 5,000 randomly selected subsets for the judgments). Note that we fitted the model after accounting for any difference in reliability between judgments and hIT. The hIT categoricality falls within the bottom 5% of the judgment distribution, which indicates that the judgments are more categorical than the hIT representation.

**Figure 12:**
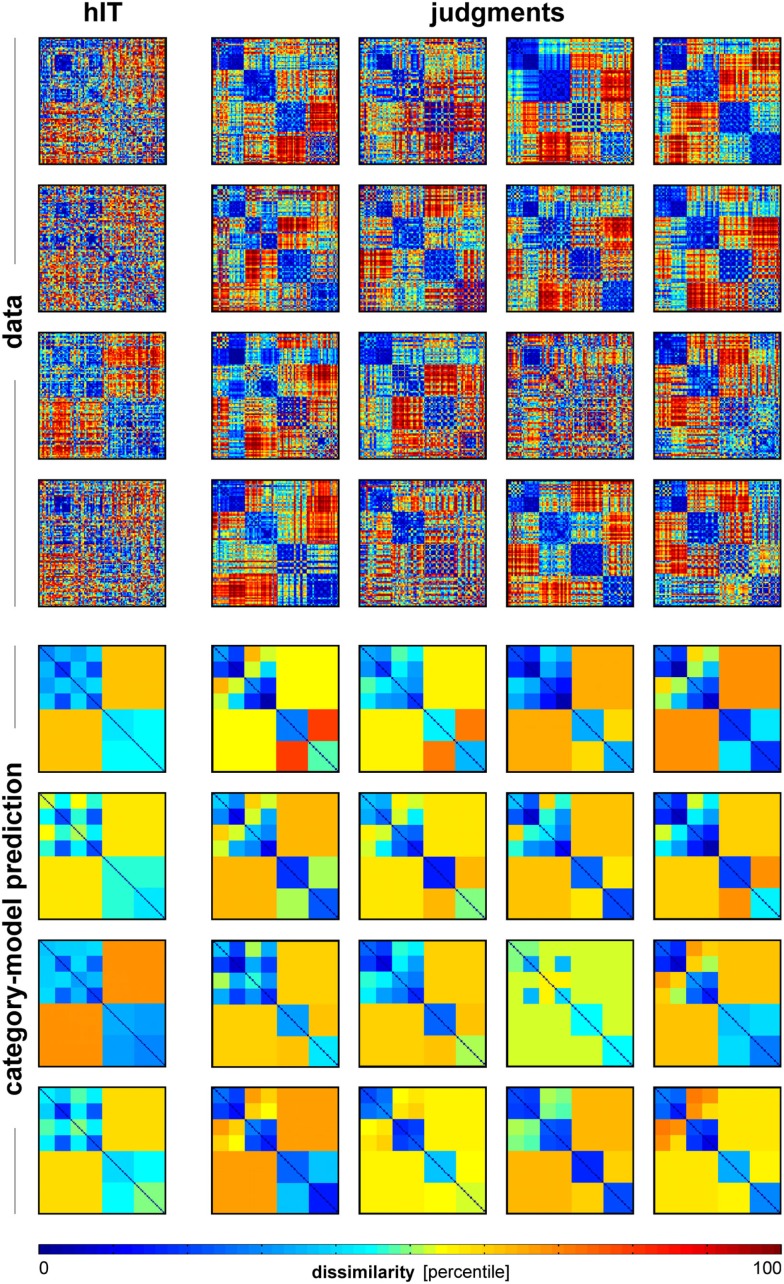
**Single-subject RDMs and category-model predictions for human IT and human judgments**. To give an impression of categoricality at the single-subject level, we plotted the single-subject RDMs for hIT and judgments (top panel), and the associated single-subject category-model predictions (bottom panel). The category-model (Figure 5B) was fitted to each subject’s RDM after equating inter-subject reliability between hIT and judgments. Visual inspection suggests stronger categoricality for the judgments than for hIT.

**Figure 13:**
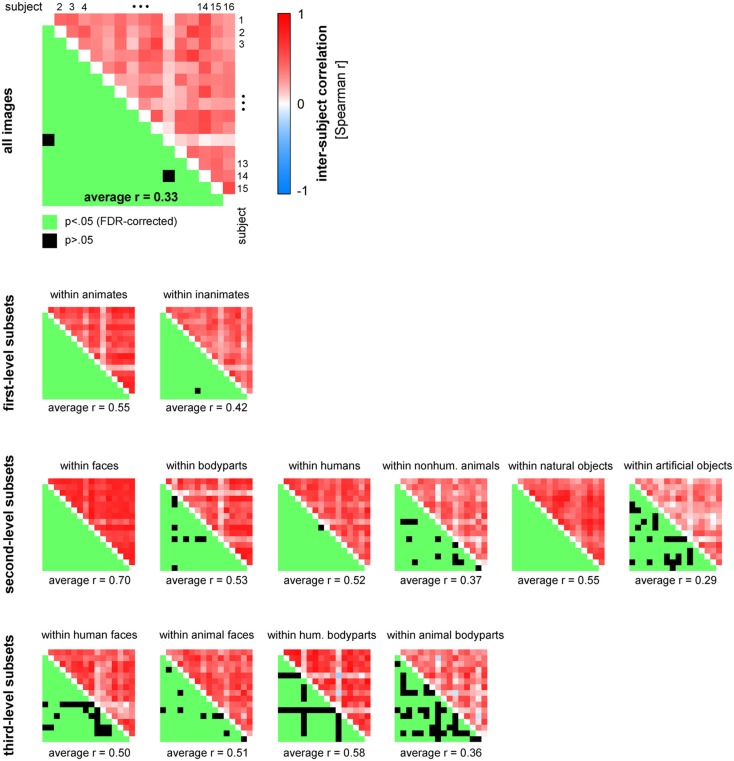
**Human similarity judgments show substantial consistency across subjects, for all images and for most category subsets of images**. The upper triangle of each matrix shows all possible pairwise inter-subject RDM correlations (Spearman *r*). The mirror-symmetric entries in the lower triangle of each matrix show the corresponding thresholded *p*-values. *p*-values were computed using a stimulus-label randomization test with 10,000 randomizations and corrected for multiple comparisons using the False Discovery Rate. The average of all pairwise 120 inter-subject correlations is shown below each matrix.

In order to investigate whether objects that elicit similar activity patterns in IT are perceived as similar, we compared dissimilarity judgments and IT activity-pattern dissimilarities for 96 color photos of isolated objects, spanning a wide range of object categories, including faces and bodies. The stimuli (Figure 1) were the same as those used in Kriegeskorte et al. (2008b) [and subset of the stimuli used in Kiani et al. (2007)]. We used the activity-pattern dissimilarity matrices estimated for human IT (hIT) and mIT in Kriegeskorte et al. (2008b). We estimated perceived dissimilarities (Figure 2) by acquiring object-similarity judgments in 16 different human observers, using a novel multi-arrangement (MA) method (Kriegeskorte and Mur, 2012), which enables efficient measurement of perceived similarity for large sets of objects. We compared the object-similarity judgments to hIT activity-pattern dissimilarities using (a) descriptive visualizations (Figures 3 and 4), (b) inferential analyses of categorical structure (Figures 5–7), (c) inferential analyses of continuous structure (Figures 8 and 9), and (d) descriptive and inferential analyses of inter-subject reliability and categoricality (Figures 10–13). We additionally related the object-similarity judgments to mIT (Figure 7), to computational models of varying complexity, and to brain-activity measurements from visual regions other than IT, including early visual cortex (EVC) (Figure 10).

## Materials and Methods

### Object-similarity judgments

#### Subjects

Sixteen healthy human volunteers (mean age = 28 years; 12 females) participated in the MA experiment. Subjects had normal or corrected-to-normal vision; 13 of them were right-handed. Before participating, the subjects received information about the procedure of the experiment and gave their written informed consent for participating. The experiment was conducted in accordance with the Ethics Committee of the Faculty of Psychology and Neuroscience, Maastricht University.

#### Multi-arrangement method

A detailed description of the MA method, including empirical validation of the method by comparison to conventional methods, can be found in Kriegeskorte and Mur (2012).

Perceived object-similarity is conventionally measured using pairwise dissimilarity judgments (e.g., Cortese and Dyre, 1996; Cooke et al., 2007). Given the large number of object pair dissimilarities to be measured in our study (96 objects, 4560 possible pairs), acquiring pairwise dissimilarity judgments, or any other measure that considers each possible pair of objects separately, would be practically difficult. Data acquisition would require many hours and multiple sessions. Moreover, subjects might change their implicit criteria when judging pairwise dissimilarities one-by-one over different sessions. The MA method solves these problems by allowing subjects to communicate multiple object-pair dissimilarities at once (Figure 2). In the MA method, subjects communicate perceived object-similarity by arranging multiple object images in 2D on a computer screen by mouse drag-and-drop. The use of spatial arrangement as a measure of perceived similarity has been proposed before (Goldstone, 1994; Risvik et al., 1994). Our MA method extends this earlier work by introducing adaptive selection of object subsets during measurement, in order to efficiently and optimally estimate perceived similarity for each individual subject. Using our MA method, the acquisition of the 4560 pairwise dissimilarities only required 1 h per subject.

The method can be summarized as follows. Each arrangement, or trial, consists of multiple (>2) objects that have to be arranged in a circular “arena” such that inter-object distances reflect perceived dissimilarity (similar objects are placed close together, dissimilar objects are placed further apart). This approach enables time-efficient measurement of perceived object-similarity because moving one object changes multiple object-pair dissimilarities at once. Single-trial estimates of object-pair dissimilarities are computed as Euclidean distances between the objects (after normalization of object positions by the diameter of the arena). On the first trial, subjects arrange all objects. On subsequent trials, they arrange subsets of objects. To optimize the object subsets to be presented on subsequent trials, we assume that the arrangements are affected by isotropic placement noise in 2D. The dissimilarity signal-to-noise ratio of the estimates then depends on how closely the objects are placed together in the arena: if two objects are placed close together (smaller dissimilarity signal), the dissimilarity estimate will have a smaller signal-to-noise ratio than when they are placed further apart. After each trial, the object subset for the next trial is constructed adaptively so as to provide more evidence for the object pairs whose current combined estimates are expected to have the greatest error, thus aiming to minimize the maximum error of the final dissimilarity estimates. For example, the object pair placed closest together on the first trial will be sampled again on the next trial so as to increase the evidence for estimating the dissimilarity of these two objects. The use of multiple trials also enables the subjects to communicate similarity relationships that would require more than two dimensions to be accurately estimated. The duration of the MA acquisition can either be fixed (e.g., 1 h as in our experiment) or contingent upon the quality of the estimated dissimilarities (e.g., ensuring that the maximum error margin across all pairs is below a certain threshold). The MA method was implemented in Matlab (The MathWorks Inc.).

We instructed our subjects to “Please arrange these objects according to their similarity,” such that similar objects were placed close together and dissimilar objects were placed further apart. The instruction intentionally did not specify which object properties to focus on, as this would have biased our perspective on the mental representation of the objects. In other words, the general instruction enabled us to investigate which properties subjects would spontaneously use when judging object-similarity for a large set of real-world object images. After performing the experiment, subjects were asked to report which object features they had used for object arrangement.

#### Construction of the representational dissimilarity matrix

For each subject, the dissimilarity estimates acquired for a given stimulus pair were averaged across trials. Rescaling of each trial’s dissimilarity estimates was required before averaging, because subjects were instructed to use the entire arena for each arrangement, making only the relations between distances on a single-trial, but not the absolute on-screen distances meaningful. For example, a given dissimilarity between two objects tended to correspond to a greater on-screen distance when the two objects appeared in a smaller subset on a given trial. The single-trial dissimilarity estimates were therefore iteratively rescaled so as to align them to the overall average (minimizing the sum of squared deviations) until convergence. The 4560 trial-average dissimilarity estimates were placed in an RDM. RDMs were constructed for each subject separately and then combined by averaging.

### fMRI experiment

Acquisition and analysis of the fMRI data have been described in Kriegeskorte et al. (2008b), where further details can be found. More information on the RSA framework can be found in Kriegeskorte et al. (2008a).

#### Subjects

Four healthy human volunteers participated in the fMRI experiment (mean age = 35 years; two females). Subjects were right-handed and had normal or corrected-to-normal vision. Before scanning, the subjects received information about the procedure of the experiment and gave their written informed consent for participating. The experiment was conducted in accordance with the Institutional Review Board of the National Institutes of Mental Health, Bethesda, MD, USA.

#### Experimental design and task

Stimuli were presented using a rapid event-related design (stimulus duration, 300 ms; interstimulus interval, 3700 ms) while subjects performed a fixation-cross-color detection task. Stimuli were displayed on a uniform gray background at a width of 2.9° visual angle. Each of the 96 object images was presented once per run. Subjects participated in two sessions of six 9 min runs each. In addition, subjects participated in a separate block-localizer experiment. Stimuli (grayscale photos of faces, objects, and places) were presented in 30-s category blocks (stimulus duration, 700 ms; interstimulus interval 300 ms). Subjects performed a one-back repetition-detection task on the images.

#### Functional magnetic resonance imaging

Blood-oxygen-level-dependent fMRI measurements were performed at high resolution (voxel volume: 1.95 mm × 1.95 mm × 2 mm), using a three Tesla General Electric HDx MRI scanner, and a custom-made 16-channel head coil (Nova Medical Inc.). We acquired 25 axial slices that covered IT and EVC bilaterally (single-shot, gradient-recalled Echo Planar Imaging: matrix size: 128 × 96, TR: 2 s, TE: 30 ms, 272 volumes per run, SENSE acquisition).

#### Estimation of single-image activity patterns

fMRI data were preprocessed in BrainVoyager QX (Brain Innovation) using slice-scan-time correction and head-motion correction. All further analyses were conducted in Matlab (The MathWorks Inc.). Single-image activity patterns were estimated for each session by voxel-wise univariate linear modeling (using all runs except those used for region-of-interest definition). The model included a hemodynamic-response predictor for each of the 96 stimuli along with run-specific motion, trend, and confound-mean predictors. For each stimulus, we converted the response-amplitude (beta) estimate map into a t map.

#### Definition of regions of interest

All regions of interest (ROIs) were defined on the basis of independent experimental data and restricted to a cortex mask manually drawn on each subject’s fMRI slices. Human IT was defined by selecting the 316 most visually responsive voxels within the IT portion of the cortex mask. Visual responsiveness was assessed using the t map for the average response to the 96 object images. The *t* map was computed on the basis of one third of the runs of the main experiment within each session. To define EVC, we selected the 1057 most visually responsive voxels, as for IT, but within a manually defined anatomical region around the calcarine sulcus within the cortex mask. The fusiform face area (FFA) (Kanwisher et al., 1997) and parahippocampal place area (PPA) (Epstein and Kanwisher, 1998) were defined based on the separate block-localizer experiment. The FFA was defined by the contrast faces minus objects and places; the PPA was defined by the contrast places minus objects and faces. Each of the four resulting unilateral regions contained 128 voxels.

#### Construction of the representational dissimilarity matrix

For each ROI, we extracted a multivoxel pattern of activity (t map) for each of the 96 stimuli. For each pair of stimuli, activity-pattern dissimilarity was measured as 1 − Pearson linear correlation across voxels within the ROI (0 for perfect correlation, 1 for no correlation, 2 for perfect anticorrelation). The resulting 4560 pairwise dissimilarity estimates were placed in an RDM. RDMs were constructed for each subject and session separately and then combined by averaging.

### Comparing representational similarity between brain and behavior

#### Descriptive visualizations

To compare hIT activity-pattern dissimilarities and dissimilarity judgments, we first visualized the data in multiple ways (Figures 3 and 4). These figures display not only the RDMs, but also the associated multidimensional scaling (MDS) plots (Torgerson, 1958; Shepard, 1980) and hierarchical cluster trees (Shepard, 1980). The MDS plots (criterion: metric stress) display the multidimensional similarity representations in 2D: the closer the objects, the more similar their activity patterns or the higher their perceived similarity. The hierarchical cluster trees (linkage method: average linking) explore, which object clusters emerge from the data when objects are grouped based on activity-pattern or perceived similarity.

#### Comparing categorical structure

The descriptive visualizations were complemented by inferential analyses addressing the question whether hIT and similarity judgments emphasize different categorical divisions. For this purpose, we assumed conventional categorical divisions (animate/inanimate, face/body, human/non-human, and natural/artificial) and tested whether the percentage of dissimilarity variance explained by a given categorical division was greater for hIT or for the similarity judgments.

##### Linear model of category-related variance

We modeled each RDM (hIT, judgments) as a linear combination of RDMs representing the category divisions and within-category clustering imbalances (Figures 5A,B). Clustering imbalance refers to a difference in degree of clustering for the categories involved in a division, e.g., stronger clustering within faces than within bodies. The model was fit using ordinary least squares. We then compared the proportion of the total dissimilarity variance explained by the category-model for hIT and judgments. This proportion was larger for the judgments (0.59) than for hIT (0.39). This must be due to a combination of two components: the within-category variance and the noise. In order to perform statistical inference on the difference between hIT and judgments with respect to a given categorical division despite the different proportions of residual variance, we took two steps. (1) We expressed variance explained by that division as a portion of the total variance explained by the category-model (thus measuring, for example, animate/inanimate variance as a percentage of total category-related variance) (Figure 5C). We used the squared beta weights to estimate explained variance, yielding estimates that are normalized for predictor energy. (2) We added dissimilarity noise to the judgment RDM, so as to equate the proportion dissimilarity variance explained by the category-model between hIT and judgments. The noise-equated judgment RDM is shown in Figure 5A.

Note that ordinary-least-squares fitting is often motivated by the fact that it gives a maximum-likelihood estimate when the errors are independent and Gaussian. Here we model dissimilarities, which are not independent or Gaussian. The ordinary-least-squares fit merely serves to give us descriptive indices of the relative strength of different categorical divisions. Our method of inference on these statistics is not dependent on assumptions of Gaussianity or independence and has been validated by simulation (An alternative approach to modeling the categorical divisions, motivated by maximum-likelihood estimation, would be to replace the correlation distances by correlations, i.e., to use similarities instead of dissimilarities, apply the Fisher *Z* transform, and then fit a category-model by least squares. The *Z* values reflecting the similarities would still be dependent and not exactly Gaussian, but perhaps the model would be preferable from a statistical perspective. This approach would require a validation study, which is beyond the scope of the present paper).

##### Equating residual variance

To equate the proportion residual variance between judgments and hIT, we assumed that the dissimilarity noise arises from isotropic Gaussian noise affecting single-subject patterns in a high-dimensional representational space. In the limit of infinite dimensions, the noise displacements are then orthogonal to the representational distances. We assumed this orthogonality and a Euclidean distance metric. By the Pythagorean theorem, for each pattern, its squared Euclidean noise displacement can then be added to each squared Euclidean distance of that pattern to other patterns, to simulate the effect of the noise. After adding the noise components to the squared Euclidean RDM, we took the square root to convert back to the original RDM units. We adjusted the standard deviation of the Gaussian noise to equate the proportion of category-related variance in the RDM.

##### Randomization test

To test whether hIT and judgments place different emphasis on a given categorical division, we performed statistical inference on the difference in percentage of explained category variance between hIT and judgments (Figure 5C). Inference was performed by randomization of the data labels (“hIT” or “judgments”) across subjects, simulating the null hypothesis that hIT and judgments do not differ in the percentages of category-related dissimilarity variance explained by the categorical divisions. We performed 10,000 randomizations, each yielding an estimate of our test statistic under the null hypothesis. If the actual difference (for the true labeling) in percentage of explained category variance for a given categorical division fell within the most extreme 5% of the simulated null distribution (two-sided test), we rejected the null hypothesis of no difference in categorical structure between hIT and judgments for that categorical division.

To check whether the label randomization test succeeds at controlling the false-positive rate at 0.05, we simulated the case of two sets of RDMs with an identical categorical structure (null hypothesis), but different levels of dissimilarity noise. The number of conditions and categorical divisions matched those in our actual data. The proportions of residual variance of the category model were set to match those in hIT and judgments. We ran the simulation 100 times, each time performing (1) the noise-adjustment step to equate the proportion of residual variance, (2) the fitting of the category model, and (3) the label-randomization test (1,000 randomizations) on the simulated RDMs. The false-positives rates for all simulated category divisions (animate/inanimate, face/body, natural/artificial) were consistently below 0.05, suggesting that our test is valid and slightly conservative.

##### Comparison between human and monkey IT

We additionally compared the categorical structure between human and mIT using the same approach (Figure 6). The monkey RDM is based on neurophysiological recordings from a population of IT cells (Kiani et al., 2007), which we previously compared to hIT in terms of continuous structure (Kriegeskorte et al., 2008b). We could not perform statistical inference for the human-monkey comparison of categorical divisions because the monkey data were based on only two subjects, which is too few to perform a valid randomization test. Instead, we compared the hIT, mIT, and human judgment RDMs in a second-order MDS arrangement (Figure 7). To test whether the RDMs were significantly related, we correlated each pair of RDMs (i.e., hIT-mIT, hIT-judgments, and mIT-judgments), and performed statistical inference on each pairwise correlation coefficient using a stimulus-label randomization test (10,000 randomizations), which simulates the null hypothesis of unrelated RDMs. If the actual correlation coefficient fell within the top 5% of the null distribution, we rejected the null hypothesis of unrelated RDMs. Even if all RDMs are significantly related, some of them might be more strongly related than others. To test whether two RDMs were more closely related than two other RDMs, we performed statistical inference on the difference of the correlation distances (1 – Pearson *r*) using bootstrap resampling of the stimulus set (1,000 resamplings). This simulates the distribution of differences between the correlation distances that we would expect to observe if we repeated the experiment for different samples of stimuli (drawn from the same hypothetical distribution of stimuli). If 0 fell in the top or bottom 2.5% of the difference distribution, we rejected the null hypothesis of equal relatedness of both pairs of RDMs and concluded that the two more highly correlated RDMs were more closely related.

#### Comparing continuous structure

We performed further inferential analyses addressing the question whether hIT and similarity judgments share continuous dissimilarity variance. To address this question, we tested whether the dissimilarity estimates of corresponding object pairs were significantly correlated between hIT and judgments. We performed this test for all objects (Figure 8), and for category subsets of objects (Figure 9). We used the same test to relate the judgments to brain-activity measurements from visual regions other than hIT, and to computational models of varying complexity (Figure 10).

We estimated the degree of correlation using Spearman’s rank correlation coefficient, since we expected a monotonic, but not necessary linear, relationship between hIT and judgment RDMs. The correlation was restricted to the lower triangle of each RDM, which contained all possible pairwise dissimilarity estimates. The classical method for inference on correlation coefficients assumes independent pairs of measurements for the variables being correlated. Such independence cannot be assumed for RDMs, because each dissimilarity estimate is dependent on two stimuli, each of which also codetermines the dissimilarities of all its other pairings in the RDM. We therefore tested the relatedness of the hIT and judgment RDMs by randomization of the stimulus labels (Figures 8A, 9A, and 10B). We performed 10,000 randomizations, each yielding an estimate of the correlation coefficient under the simulated null hypothesis that hIT and judgments do not share continuous dissimilarity variance. The obtained estimates served as a null distribution for statistical inference. If the actual correlation coefficient fell within the top 5% of the simulated null distribution, we rejected the null hypothesis of unrelated RDMs.

We also tested the relatedness of the hIT and judgment RDMs in a random-effects analysis across subjects (Figures 8B and 9B). This analysis enables generalization of the results to the population and does not assume independence of the dissimilarity estimates in an RDM. We first computed single-subject Spearman rank correlation coefficients by correlating each single-subject judgment RDM with the subject-average hIT RDM. We then transformed these correlation coefficients using the Fisher *Z* transform and performed a standard one-sample *t* test on the resulting *Z* values. The *t* test was used to determine whether the average of the single-subject *Z* values was larger than zero.

Measurement noise affects correlation estimates, e.g., it might weaken the observed correlation between two variables (hIT, judgments). An attenuation correction could alleviate the influence of noise, however, this would ideally require estimating the test-retest reliability of the hIT and judgment data. This was not feasible since the judgments were acquired in a single session. The reported correlation coefficients are therefore not corrected for attenuation. Although this might have decreased our sensitivity to effects, it does not affect the validity of our stimulus-label randomization test.

#### Comparing inter-subject reliability and categoricality

To get an impression of the inter-subject variability of hIT and judgment RDMs, we performed second-order MDS (criterion: metric stress; distance measure: 1 − Spearman *r*) on the single-subject RDMs for hIT and judgments combined (Figure 11A): the closer two subjects in the MDS plot, the more similar their representational similarity structures. The MDS visualizations were complemented by inferential analyses addressing the question whether hIT and judgments differ in inter-subject reliability (Figure 11B). We estimated inter-subject reliability as the average pairwise correlation (Spearman *r*) between single-subject RDMs. We first computed the inter-subject reliability for hIT (four subjects, six pairwise comparisons), and then repeatedly selected random subsets of four subjects from the judgment data (16 subjects) to estimate inter-subject reliability for the judgments (5,000 randomizations). We used these 5,000 estimates as a null distribution for statistical inference: if the hIT estimate fell within the most extreme 5% of the judgment distribution, we rejected the null hypothesis of no difference between hIT and judgments in inter-subject reliability.

If we consider both measurement error and inter-subject variation as noise, we can equate noise levels by equating inter-subject reliability, and address the question whether the similarity judgments are more categorical than the hIT representation (Figure 11C). Although inter-subject reliability was not significantly different between judgments and hIT, we explicitly equated it using the same procedure as described previously under the heading ‘Equating residual variance’ (i.e., by adding dissimilarity noise to the single-subject judgment RDMs; inter-subject reliability for hIT was 0.28, average inter-subject reliability for the judgments was 0.32). Note that this time the amount of noise was adjusted to equate inter-subject reliability, not the proportion of category-related variance, enabling us to compare the latter between judgments and hIT. We fitted the category model (shown in Figures 5 and 6) to the noise-equated single-subject RDMs, and computed the proportion of category-related dissimilarity variance for each subject. We used the subject-average proportion of category-related dissimilarity variance as an estimate of categoricality. We first estimated categoricality for hIT (four subjects), and then repeatedly selected random subsets of four subjects from the judgment data (16 subjects) to estimate categoricality for the judgments (5,000 randomizations). We used these 5,000 estimates as a null distribution for statistical inference: if the hIT estimate fell within the bottom 5% of the judgment distribution, we rejected the null hypothesis of no difference between hIT and judgments in categoricality. Figure 12 shows the single-subject hIT and judgment RDMs and the single-subject category-model predictions (estimated using noise-equated RDMs) for visual inspection.

Figure 13 displays inter-subject reliability for the judgments in more detail. The correlation matrices show all possible pairwise inter-subject correlation coefficients (Spearman *r*), for all images (top panel), and for category subsets of images (smaller panels). Statistical inference was performed using stimulus-label randomization tests, simulating the null hypothesis of uncorrelated RDMs. Results were corrected for multiple comparisons using the False Discovery Rate.

### Model representations of the stimuli

We processed our stimuli to obtain their representations in a number of simple and complex computational models. The model representations have been described previously (Kriegeskorte et al., 2008a,b), but are repeated here for completeness. Each image was converted to a representational vector as described below for each model. Each representational vector was then compared to each other representational vector by means of 1-*r* as the dissimilarity measure (where *r* is Pearson correlation coefficient). The resulting model RDMs were then compared to the similarity judgment RDM (Figure 10).

#### Binary silhouette image

The RGB color images (175 × 175 pixels) were converted to binary silhouette images, in which all background pixels had the value 0 and all figure pixels had the value 1. Each binary silhouette image was then converted to a pixel vector (175 × 175 binary numbers).

#### Luminance image

The RGB color images (175 × 175 pixels) were converted to luminance images. Each luminance image was then converted to a pixel vector (175 × 175 numbers).

#### Color image (CIELAB)

The RGB color images (175 × 175 pixels) were converted to the CIELAB color space, which approximates a linear representation of human perceptual color space. Each CIELAB image was then converted to a pixel vector (175 × 175 × 3 numbers).

#### Color set (joint CIELAB histogram)

The RGB color images (175 × 175 pixels) were converted to the CIELAB color space. The three CIELAB dimensions (*L*, *a*, *b*), were then divided into six bins of equal width. The joint CIELAB histogram was computed by counting the number of figure pixels (gray background left out) falling into each of the 6 × 6 × 6 bins. The joint histogram was converted to a vector (6 × 6 × 6 numbers).

#### V1 model

The luminance images (175 × 175 pixels, 2.9° visual angle) were given as input to a population of modeled V1 simple and complex cells (Riesenhuber and Poggio, 2002; Lampl et al., 2004; Kiani et al., 2007). The receptive fields (RFs) of simple cells were simulated by Gabor filters of four different orientations (0°, 90°, −45°, and 45°) and 12 sizes (7–29 pixels). Cell RFs were distributed over the stimulus image at 0.017° intervals in a cartesian grid (for each image pixel there was a simple and a complex cell of each selectivity that had its RF centered on that pixel). Negative values in outputs were rectified to zero. The RFs of complex cells were modeled by the MAX operation performed on outputs of neighboring simple cells with similar orientation selectivity. The MAX operation consists in selecting the strongest (maximum) input to determine the output. This renders the output of a complex cell invariant to the precise location of the stimulus feature that drives it. Simple cells were divided into four groups based on their RF size (7–9 pixels, 11–15 pixels, 17–21 pixels, 23–29 pixels) and each complex cell pooled responses of neighboring simple cells in one of these groups. The spatial range of pooling varied across the four groups (4 × 4, 6 × 6, 9 × 9, and 12 × 12 pixels for the four groups, respectively). This yielded 4 (orientation selectivities) × 12 (RF sizes) = 48 simple-cell maps and 4 (orientation selectivities) × 4 (sets of simple-cell RF sizes pooled) = 16 complex cell maps of 175 × 175 pixels. All maps of simple and complex cell outputs were vectorized and concatenated to obtain a representational vector for each stimulus image. We also included a version of the V1 model in which we averaged all simple and complex cell responses representing the same retinal location (averaging also across orientation selectivities and RF sizes) in order to mimic the effect of downsampling by population averaging within fMRI voxels (“V1 model, smoothed”).

#### HMAX-C2 based on natural image fragments

This model representation developed by Serre et al. (2005) builds on the complex cell outputs of the V1 model described above (implemented by the same group). The C2 features used in the analysis may be comparable to those found in primate V4 and posterior IT. The model has four sequential stages: S1-C1-S2-C2. The first two stages correspond to the simple and complex cells described above, respectively. Stages S2 and C2 use the same pooling mechanisms as stages S1 and C1, respectively. Each unit in stage S2 locally pools information from the C1 stage by a linear filter and behaves as a radial basis function, responding most strongly to a particular prototype input pattern. The prototypes correspond to random fragments extracted from a set of natural images (stimuli independent of those used in the present study). S2 outputs are locally pooled by C2 units utilizing the MAX operation for a degree of position and scale tolerance. A detailed description of the model (including the parameter settings and map sizes we used here) can be found in Serre et al. (2005). The model, including the natural image fragments, was downloaded from the author’s website in January 2007 (for the current version, see http://cbcl.mit.edu/software-datasets/standardmodel/index.html).

#### RADON transform

As an example of a model inspired by image processing, we included the Radon transform, which has been proposed as a functional account of the representation of visual stimuli in the lateral occipital complex. The Radon transform of a two-dimensional image is a matrix, each column of which corresponds to a set of integrals of the image intensities along parallel lines of a given angle. We used the Matlab function radon to compute the Radon transform for each luminance image. We additionally used smoothed versions of these radon-transformed images (low-passed), which were computed by convolving the transformed images with a Gaussian kernel of 11.75 pixels full width at half maximum (“radon, smoothed”).

## Results

### Human judgments reflect IT categorical divisions and introduce human-related additional divisions

Figure 3 visualizes the dissimilarity data for judgments and hIT. Both the RDMs (Figure 3A) and MDS arrangements (Figure 3B) suggest that the human judgments strongly emphasize conventional categorical divisions. The top-level division is animate/inanimate just like in the hIT representation. In addition, both hIT and judgments show a tight cluster of human faces. Compared to the hIT representation, the judgments appear to exhibit tighter (sub)clusters, which could reflect the nature of the representation or different noise levels of the measurements. Further analyses support the first explanation (Figure 11). The cluster analysis (Figure 4) suggests that, in addition to the animate/inanimate and face/body divisions that are present in both representations, the judgments show a natural/artificial division among the inanimate objects and a prominent human/non-human division among the animate objects. The human/non-human division appears at a higher level of the hierarchy (suggesting that it explains more pattern variance) than the face/body division. Both additional divisions observed in the human judgments concern the distinction between human-associated objects (human face or body, or artificial, i.e., man-made, object) and non-human-associated objects (non-human face or body, or natural object).

Debriefing reports of the subjects are consistent with the descriptive visualizations of the judgment data. Fifteen out of sixteen subjects indicated that they arranged the objects by semantic category. The specific categories mentioned by the subjects correspond to the (sub)clusters shown in Figure 3B (e.g., human faces, monkeys/apes, fruits, tools). Most subjects indicated that they also used shape and color to arrange the objects, specifically within category clusters.

Figure 5 shows the inferential analysis of category-related variance components. The categories were defined according to our prior hypotheses based on the literature and used conventional categorical divisions (animate/inanimate, face/body, human/non-human, natural/artificial). We used a linear model of category-related dissimilarity variance (Figures 5A,B) and estimated the percentage of the total category-related variance explained by each categorical division (Figure 5C). Consistent with the clustering results, this showed that the animate/inanimate and face/body divisions were prominent in both hIT and judgments, and that the judgments additionally introduced the divisions human/non-human and natural/artifical. Inferential comparisons showed that the human/non-human and the natural/artificial division are significantly stronger in the judgments than in hIT (*p* < 0.01 for both divisions, randomization test), and the animate/inanimate and the face/body division are significantly weaker (*p* < 0.01, *p* < 0.025, respectively). Since the category-related variance claimed by each division is defined as a percentage of the total category-related variance for each RDM, the additional divisions seen in the judgments come at the expense of the other divisions. The smaller percentage for the animate/inanimate and the face/body division in the judgments might, thus, be entirely explained by the additional divisions.

### Human IT is more closely related to monkey IT than to human judgments

Figures 6 and 7 bring in the mIT data. Figure 6 suggests that hIT and mIT share their major categorical divisions, i.e., the top-level animate/inanimate division and the face/body division within the animates, consistent with descriptive visualizations in earlier work (Kriegeskorte et al., 2008b). Figure 7 visually and inferentially relates the three RDMs (hIT, mIT, and human judgments). The three RDMs are significantly related, as are their category-related components of the dissimilarity variance (*p* < 0.0001 for each pairwise comparison). This, however, does not exclude that some RDMs might be more strongly related than others. Further analyses showed that the RDMs for hIT and mIT are significantly more similar than either of them is to the judgments (*p* < 0.05, *p* < 0.01, respectively; Figure 7A). When we consider only the category-related component of the dissimilarity variance (Figure 7B), this effect is even more pronounced: hIT and mIT are much more similar to each other than either of them is to the judgments (*p* < 0.001, *p* < 0.001, respectively). When we consider only the non-category-related component (Figure 7C), we see a weak reflection of the same qualitative picture: hIT and mIT appear slightly more similar to each other than either of them is to the judgments (*p* > 0.05, *p* < 0.01, respectively). Consistent with this finding, the non-category-related components are significantly correlated between human and monkey IT (*p* < 0.0001) but not between human judgments and either monkey or human IT. We did not find any evidence that human judgments are more closely related to hIT than to mIT for the original RDMs, for the category-related component RDMs, or for the non-category-related component RDMs.

### Human judgments reflect the IT representation, even within categories

We have seen that hIT dissimilarities are more closely related to mIT dissimilarities than to human dissimilarity judgments. This does not mean that human judgments do not reflect the hIT representation. To investigate in detail to what extent and for which categories hIT and judgments share continuous dissimilarity variance, we tested whether the dissimilarities were significantly correlated between hIT and judgments across object pairs. We performed this test for all objects (in which case a significant correlation could be driven by shared categorical divisions) and for category subsets of objects. The dissimilarities were significantly correlated, both within all objects and within most category subsets of objects (Figures 8A and 9A). In particular, dissimilarities were significantly correlated (stimulus-label randomization test applied to group-average RDMs) within the following categories: animate objects, inanimate objects, faces, bodies, human bodies, humans (faces and bodies), non-human animates (faces and bodies), natural objects, and artificial objects. We found no evidence for a dissimilarity correlation between hIT and human judgments within the following categories: human faces, animal faces, and animal bodies. The highest correlation coefficients between hIT activity-pattern dissimilarities and dissimilarity judgments were found within humans (*r* = 0.60), within faces (*r* = 0.40), and within natural objects (*r* = 0.46).

A similar pattern of dissimilarity correlations between hIT and human judgments was found in a random-effects analysis across subjects (Figures 8B and 9B). Again, hIT and judgments were significantly correlated within all images and within most category subsets of images, including all subsets that were identified by the stimulus-label randomization test. This suggests that our results can be generalized to the population of similarity-judgment subjects. These results show that, although judgments emphasize additional categorical divisions, they do reflect the representational dissimilarities of IT, even within categories.

### Human IT explains human judgments better than other ventral-stream regions and computational models

Other brain regions, including EVC, the FFA, and the PPA, did not match the judgments as well as hIT (Figure 10). FFA showed a lower, but still significant correlation with the similarity judgments (*r* = 0.22, *p* < 0.0001); for EVC and PPA, the correlation was not significant. Computational models based on low-level and more complex natural image features also did not match the similarity judgments as well as hIT (Figure 10B). Among the models, simple models based on object color and shape, and a more complex model based on natural image features thought to be representative of primate V4 and posterior IT (Serre et al., 2005), showed the closest match to the similarity judgments.

### Human judgments show stronger categoricality than human IT

Categorization is a hallmark of human judgment, so one might expect judgments to be more categorical than the high-level visual representation. The MDS arrangement in Figure 3B and the hierarchical clustering trees in Figure 4 might seem to support this prediction, suggesting that judgments are more strongly categorical than hIT. However, this appearance could have resulted from more noise in the hIT measurements. We therefore inferentially compared the reliability of hIT and judgment RDMs and also inferentially compared categoricality after accounting for any difference in reliability. Results are shown in Figure 11. The MDS arrangement of single-subject RDMs in Figure 11A shows that subjects cluster according to type of measurement (judgments or hIT), but also suggests similar variability across subjects for judgments and hIT. Consistent with this observation, Figure 11B shows that inter-subject reliability does not differ significantly between hIT and judgments (randomization test, two-sided *p* = 0.61), i.e., the judgment and hIT measurements are equally reliable. We then tested whether the judgments are more categorical than the hIT representation by comparing the proportion of category-related dissimilarity variance between hIT and judgments (Figure 11C). The subject-average proportion of category-related variance was 0.21 for hIT, and 0.31 for the judgments (the value reported for the judgments is the mean of the judgment distribution, see Materials and Methods). The results of our test suggest that the judgments are indeed more categorical than the IT representation (randomization test, one-sided *p* < 0.05). Visual inspection of Figure 12, which displays all single-subject RDMs and category-model predictions, is consistent with this conclusion: it gives the impression of stronger categoricality of the judgments than hIT, even at the single-subject level. Together, these results suggest that the strong categorical clustering observed for the judgments in Figures 3 and 4 reflects a difference in the nature of the two representations, not a difference in measurement noise.

### Human judgments show substantial consistency across subjects

Figure 10A shows that the single-subject judgment RDMs cluster together within the larger context provided by the RDMs of different brain regions and computational models. One of the subjects (S11) falls outside of the cluster, showing a similarity representation more similar to simple models based on image features than to the similarity representations of the other subjects. This subject reported to have arranged objects by shape instead of semantic category. Consistent with the observation that single-subject representations cluster together, all but two of the 120 possible pairwise correlations between single-subject RDMs were significantly greater than zero (Figure 13, top panel). These results could be driven (completely) by category divisions shared across subjects. We therefore repeated the same procedure for category subsets of images (Figure 13, smaller panels). Results suggest that, for most tested categories, within-category similarity structure is also shared across subjects.

## Discussion

### Human object-similarity judgments are categorical and reflect the IT object representation

We asked subjects to judge object-similarity for a large set of real-world object images and investigated whether these similarity judgments reflected the IT object representation, including its hierarchy of category clusters and within-category structure. Our results show that human similarity judgments are categorical (consistent with Rosch et al., 1976; Edelman et al., 1998) and reflect the two major categorical divisions that characterize the primate-IT object representation: the top-level animate/inanimate division and the face/body division among the animates (Kiani et al., 2007; Kriegeskorte et al., 2008b).

The shared top-level animate/inanimate division relates to neuropsychological (Warrington and Shallice, 1984; Capitani et al., 2003), behavioral (Kirchner and Thorpe, 2006; New et al., 2007), and neuroimaging findings (Martin et al., 1996; Chao et al., 1999; Kriegeskorte et al., 2008b; Connolly et al., 2012; Naselaris et al., 2012) that suggest a special status for the living/non-living division. This special status might be explained in terms of evolutionary pressure toward fast and accurate recognition of animals (New et al., 2007). Recognizing animals, whether they were predator or prey, friend or foe, was of vital importance to our primate ancestors. Recognizing faces was key to survival and reproduction as well, since faces carry important information that can be used to infer the emotions, intentions, and identity of other animals. The IT representation is likely to play a central role in these essential visual functions, and might be optimized, at the phylo- and/or ontogenetic level, to distinguish essential categorical divisions.

Alternatively, one might argue that the categorical structure of both the similarity judgments and the IT object representation can be explained in terms of visual similarity. We refer to features as “visual” if they are not expressly designed (e.g., by supervised learning) to discriminate categories or encode semantic variables. Previous studies have shown a relationship between perceived visual shape similarity and IT activity-pattern similarity for abstract object shapes (Kayaert et al., 2005; Haushofer et al., 2008; Op de Beeck et al., 2008). Animate and inanimate objects differ in the parts they are composed of and consequently in visual properties (Tyler and Moss, 2001). For sufficiently visually distinct categories, category clustering is expected to arise solely based on visual similarity. In order to test if our findings could be accounted for by visual similarity, we studied model representations of the stimuli. A simple silhouette model and a more complex computational model based on natural image features at a level of complexity thought to approximately match V4 and posterior IT (Serre et al., 2005) – do not show a clear categorical structure (Figure 10; for more detailed analyses, see Kriegeskorte et al., 2008b), and do not account for either the similarity judgments or IT. We are in the process of testing a wider range of models. It is important to note that the space of visual feature representations that could be considered is infinite, and so a visual feature account can never strictly be ruled out. However, our current interpretation is that the IT features might be designed to emphasize behaviorally important categorical divisions.

It has been shown that visual features of intermediate complexity, which IT is sensitive to (Tanaka, 1996), are optimal for category discrimination (Ullman et al., 2002). However, sensitivity to visual features of intermediate complexity alone does not lead to a categorical object representation. What may be needed is explicit design, i.e., selection of the visual features that are most informative about category membership (Ullman et al., 2002). Indeed, some studies have suggested that IT is especially sensitive to category-discriminating visual features (Sigala and Logothetis, 2002; Lerner et al., 2008). Categories whose detection is highly important to the organism, including animals and faces (see also Mahon et al., 2009), are most likely to be represented by optimized IT features (Schyns et al., 1998).

Our results show that similarity judgments reflect not only the two major categorical divisions of the IT representation, but also the IT within-category similarity structure. Given the functional properties of IT, this within-category match is likely to be based on visual similarity between objects that belong to the same category cluster. This explanation is consistent with the reports of our subjects, stating that they used object color and shape to arrange objects within category clusters. Furthermore, these findings are consistent with the previously reported relationship between perceived object shape and IT activity-pattern similarities (Edelman et al., 1998; Kayaert et al., 2005; Haushofer et al., 2008; Op de Beeck et al., 2008). The matching within-category dissimilarities of IT and judgments might also be explained in terms of a common underlying prototype model (see Cutzu and Edelman, 1998; Edelman, 1998).

### Human object-similarity judgments transcend the primate-IT object representation

Several features of the object-similarity judgments cannot be explained by the IT representation. The human judgments show stronger categorical clustering and introduce additional human-specific categorical divisions: between human and non-human animals and between man-made and natural objects. Both of these additional divisions relate to the human species. They could reflect the involvement of other brain systems that either contribute these particular divisions or enable flexible task-dependent categorization.

It is important to note that judging similarity is a complex conscious process associated with shifts of overt and covert attention, while the IT representation was characterized here under passive-viewing conditions, while the subjects performed a task at fixation to which the objects were irrelevant. Our finding that IT, under passive-viewing conditions, predicts some of the major categorical divisions and within-category structure in the similarity judgments suggests an involvement of IT in the judgments. However, the nature of the judgment task is such that it will involve many brain systems, including those associated with attention and executive control, and these might even influence the representational space within IT. These brain systems might include prefrontal cortex, which has been implicated in goal-directed behavior (see Duncan, 2010) and task-dependent categorization (Freedman et al., 2001; Roy et al., 2010; but see Minamimoto et al., 2010).

Similarity judgments are dependent on task instruction (Liu and Cooper, 2001). The task instruction given to the subjects in our experiment was very general (“Please arrange these objects according to their similarity”). Note that the instruction did not refer to “images,” but to “objects” and thus presumably elicited functional and semantic descriptions along with visual ones. Each object can be described by multiple properties, including color, shape, real-world size, function, and semantic category, and subjects were free to choose and weight these properties according to their subjective preferences. Nevertheless, subjects exhibited a strong tendency to group the objects by the same four semantic categories (human, non-human animal, natural object, artificial object) and by similar within-category features. The consistency across subjects may reflect the shared component of the human experience. The focus on these categorical divisions makes sense given their importance in daily life.

From the comparison here of IT and judgments within humans, it is clear that the human judgments introduce additional divisions. We did not have comparable behavioral data for the monkeys, so we do not know whether monkeys also introduce additional divisions when judging similarity in the context of natural behavior. Previous behavioral studies in monkeys showed that monkeys represent objects categorically (Sands et al., 1982; Kiani et al., 2007), but these studies did not investigate differences in categorical structure between IT activity-pattern similarity and perceived similarity.

### Future directions

Our study takes an important first step toward the identification of the neuronal basis of similarity judgments of real-world object images. Our focus here was on the ventral-stream object representation. Future research should investigate the similarity representation in the entire brain, for example using a searchlight mapping approach (Kriegeskorte et al., 2006) to find the region that matches the similarity judgments most closely. A closer match to the similarity judgments might also be found by combining information from different brain regions.

Another avenue for future research would be to systematically investigate the effect of task instruction on both the judgments and the brain representation. Task instruction can be used to “bias” the subjects toward using certain object dimensions for judging object-similarity, e.g., color, shape, real-world size, esthetic appeal. It will be interesting to see to what degree the similarity judgments reflect the task instruction and how task instruction modulates the explanatory contributions of different brain regions. Furthermore, the influence of task instruction on inter-subject consistency could be investigated. A more specific task instruction might increase inter-subject consistency, but this might also depend on the object property mentioned in the task instruction (e.g., color vs. esthetic appeal).

One drawback of the current study is that the judgments and the IT representation are based on different groups of subjects. This enabled a more interpretable comparison of the explanatory power of the IT representation in human and monkey, and does establish a close relationship between judgments and IT. However, it is important to also investigate brain representations and judgments in the same subjects (e.g., Haushofer et al., 2008; Op de Beeck et al., 2008). This might reveal an even closer match and might enable us to explain individual idiosyncrasies of the judgments on the basis of the same subjects’ brain representations.

### Conclusion

We conclude that human similarity judgments of visually presented real-world objects reflect the categorical divisions that are prominent in the primate-IT representation and also the within-category similarity structure of the IT representation. The IT categorical divisions include animate/inanimate and face/body, divisions that are behaviorally important to all primates. Despite reflecting IT, similarity judgments also transcend the IT representation in that they introduce additional categorical divisions. In the human, these are the human-specific distinctions between humans and other animals and between man-made and natural objects. These divisions unexplained by IT may reflect a contribution to similarity judgments from other brain systems that enable flexible categorization for adaptive behavior.

## Conflict of Interest Statement

The authors declare that the research was conducted in the absence of any commercial or financial relationships that could be construed as a potential conflict of interest.
